# Radical gastrectomy is safe for treatment of gastric cancer patients on immunosuppressive drugs after organ transplantation

**DOI:** 10.3389/fonc.2023.1264628

**Published:** 2024-01-10

**Authors:** Jin Ah Lee, So Jung Kim, Ho Seok Seo, Han Hong Lee, Sung Geun Kim, Kyong Hwa Jun, Kyo Young Song, Yoon Ju Jung

**Affiliations:** ^1^ Division of Gastrointestinal Surgery, Department of Surgery, Seoul St. Mary’s Hospital, College of Medicine, The Catholic University of Korea, Seoul, Republic of Korea; ^2^ Division of Gastrointestinal Surgery, Department of Surgery, Yeouedo St. Mary’s Hospital, College of Medicine, The Catholic University of Korea, Seoul, Republic of Korea; ^3^ Division of Gastrointestinal Surgery, Department of Surgery, St. Vincent’s Hospital, College of Medicine, The Catholic University of Korea, Suwon, Republic of Korea

**Keywords:** gastric cancer, organ transplantation, immunosuppressive drug, gastrectomy, diagnosis, prognosis

## Abstract

**Background:**

*De novo* malignancies are major causes of death after organ transplantation because the recipients subsequently receive immunosuppressant drugs. When gastric cancer develops, the clinical course of the tumor may be particularly aggressive. However, there are few reliable studies of gastric cancer treatment after organ transplantation. This study examined the clinicopathological characteristics of gastric cancer patients after organ transplantation and evaluated treatment outcomes after gastrectomy.

**Methods:**

Clinical data were collected from 54 patients who were diagnosed with gastric cancer after organ transplantation. Of these, 30 who underwent surgery for gastric cancer while on immunosuppressant medications were compared with a control group of 625 gastric cancer patients. To compensate for clinical differences between the two groups, 1:1 propensity-score matching was performed.

**Results:**

Among the 30 gastric cancer patients on immunosuppressants, kidney transplantation was the most common procedure (19/30, 63.3%) followed by bone marrow (6) and liver transplantation (4); among all 54 patients, 45 were on one or two immunosuppressants. Up-migration to an advanced pathological stage was more frequent in the transplant group. In multivariate analysis, transplantation was a significant risk factor for up-migration from the T, M, and final stages after surgery. When the 30 patients on immunosuppressants who underwent gastric cancer surgery were compared with the matched controls, the total incidence (30.0 *vs* 40.0%, *P* = 0.417) and the number of severe postoperative complications (16.7 *vs* 13.4%, *P* = 0.417) did not differ significantly between groups after propensity score matching. In terms of overall survival, the transplant group showed significantly worse prognosis in stages I, II, and IV (*P* < 0.001*, P* = 0.039 and 0.007, respectively).

**Conclusion:**

Radical gastrectomy can be a safe oncological procedure for gastric cancer patients on immunosuppressants after transplantation. Considering their immunosuppressed condition and the possibility of underestimation of the stage of gastric cancer, early detection with endoscopic screening is needed to allow curative treatment.

## Introduction

The survival rate of transplant patients is increasing with the use of powerful immunosuppressive agents. As survival rates increase, the cumulative doses of these agents also increase (1, 2). This is good news for the survival of the grafted organ. However, these agents inevitably suppress the patient’s immune system, reducing the ability of the immune surveillance system to suppress cancer development or the proliferation of cancer-related viruses (3, 4). The cumulative immunosuppressant dose is an important risk factor for cancer, and combination therapy also increases the risk of cancer (5).

In a surveillance report focused on a Korean population in 2006, 105 of 1500 (7.1%) kidney transplant patients subsequently developed *de novo* cancer (6). Gastric cancer was the most common cancer type, at 16.2%, and the mean interval from transplantation to cancer development was approximately 10 years. When further follow-up data were published, 141 of 1700 patients had post-transplant cancer, and gastric cancer remained the most common type (7). Older age, female sex, graft function duration, and follow-up duration have been identified as risk factors for cancer (7). However, little is known about the clinical outcomes of the gastric cancer and postoperative safety in this situation. Therefore, this study examined the clinicopathological characteristics and surgical safety of gastric cancer patients after organ transplantation.

## Patients and methods

### Study populations

This study reviewed the medical records of gastric cancer patients who had undergone radical gastrectomy with curative intent at Seoul St. Mary’s Hospital and St. Vincent Hospital between 1990 and 2021; the review identified 54 patients who had undergone transplantation before the diagnosis of gastric adenocarcinoma. Because of the large difference in the number of patients, we limited the control group to gastric cancer patients who underwent surgery between 2015 and 2018 in Seoul St. Mary’s Hospital. After the exclusion of patients who were treated with conservative care or endoscopic submucosal dissection without radical gastrectomy, or patients who were taking no immunosuppressant because of graft failure or acquired tolerance after bone marrow transplant, 30 patients on immunosuppressants after transplantation were compared with the control group of gastric cancer patients. **Supplementary Figure 1** shows the algorithm for patient inclusion and exclusion. There was a change in the cancer staging system used for 30 years from the 1990s, and it was updated from the 4th to the 8th edition of American Joint Committee on Cancer, but all staging was redefined to the 8th edition for present analysis. Patients underwent conventional radical gastrectomy with curative intent in accordance with the Japanese (8–10) and Korean Gastric Cancer Treatment Guidelines (11). Postoperative complications were defined as any unwanted event during surgery or the patient’s condition within 30 days after surgery. The severity of complications and reinterventions was graded using the Clavien–Dindo classification (grades I–V), and a serious complication was defined as Clavien–Dindo grade III or higher. Pathological stage was classified according to the 8^th^ American Joint Committee on Cancer criteria. This study was approved by the Institutional Review Board of the College of Medicine, Catholic University of Korea (KC22RISI0943). Patient records were anonymized and deidentified before analysis.

### Statistical analysis

SPSS for Windows (ver. 22.0; SPSS, Chicago, IL, USA) was used to perform the statistical analyses. The chi-squared and Fisher’s exact tests were used to evaluate the associations among categorical variables, whereas Student’s *t*-test was used for continuous variables. Various factors, such as the Eastern Cooperative Oncology Group (ECOG) score, body mass index (BMI), comorbidity, smoking history, and alcohol history, were analyzed through 1:1 propensity score matching. Survival analysis was performed using the Kaplan–Meier method. All results were considered statistically significant when *p*-values were < 0.05.

## Results

**Supplementary Table 1** summarizes the clinicopathological characteristics of the 54 patients who had undergone transplantation. The mean patient age was 58.25 ± 11.47 years, and 39 patients (72%) were men. Of the 54 patients, 24 (44.44%) had clinically early cancer. Kidney transplantation was the most common organ transplant (39 of 54, 72.22%); other types of transplants were liver, bone marrow, and heart. Of the 54 patients, 45 (83.33%) were on immunosuppressant agents, including 19 (35.1%) who were taking two or more agents. Nine patients discontinued immunosuppressants (**Supplementary Figure 1**), including two who resumed hemodialysis due to graft failure after kidney transplantation, six who did not require immunosuppressants due to acquisition of tolerance after bone marrow transplantation, and one whose leukemia relapsed after bone marrow transplantation.

The patients’ preoperative characteristics and postoperative outcomes were compared between the 30 patients who underwent gastrectomy after transplantation (TP group) and a control group of gastric cancer patients (non-TP group, **Tables 1** and **2**). The mean age and proportion of men did not significantly differ between the two groups; the TP group had a significantly lower mean BMI (20.87 kg/m^2^ *vs*. 23.76 kg/m^2^, *p* < 0.001) and higher ECOG score (*p* < 0.001). Cancer location and clinical stages did not significantly differ between the groups (**Table 1**). Patients in the non-TP group underwent more distal gastrectomy (*p* < 0.001) with D2/D2+ lymph node dissection (*p* < 0.013), but the operating time and estimated blood loss were not significantly different. Significantly fewer lymph nodes were retrieved in the TP group (22.21 *vs*. 43.98, *p* < 0.001), and the pathological stages were significantly advanced in the TP group (pT, N, M, and final stage, all *p* < 0.001). Postoperative complication rates did not significantly differ between the two groups (**Table 2**).

**Table 1:** Preoperative clinical characteristics of the patients who underwent gastric cancer surgery after transplantation and under immunosuppressant.

		TP group N=30	None TP group N= 625	P-value
Age		59.23 (±12.62)	61.60 (±11.71)	0.281
Sex	Male	20 (66.7%)	403 (64.5%)	0.807
	Female	10 (33.3%)	222 (35.5%)	
BMI		20.87 (±3.24)	23.76 (±3.29)	< 0.001
PS (ECOG)	0	5 (16.7%)	504 (80.6%)	< 0.001
	1	22 (73.3%)	101 (16.2%)	
	2	2 (6.7%)	17 (2.7%)	
	3	2 (6.7%)	3 (0.5%)	
Comorbidity	Hypertension	18 (60.0%)	218 (34.9%)	0.005
	DM	16 (53.3%)	90 (14.4%)	< 0.001
	Pulmonary	2 (6.7%)	39 (6.2%)	0.925
	others	29 (96.7%)	272 (43.5%)	< 0.001
Smoking	None	27 (90.0%)	241 (38.6%)	< 0.001
	Ex-smoker	2 (6.7%)	196 (31.4%)	
	Current smoker	1 (3.3%)	188 (30.1%)	
Alcohol	None	29 (96.7%)	242 (38.7%)	< 0.001
** **	Social drinking	1 (3.3%)	248 (39.7%)	
** **	Heavy drinking	1 (0%)	135 (21.6%)	
Cancer location	Upper 1/3	4 (13.3%)	96 (15.4%)	0.549
** **	Middle 1/3	5 (16.7%)	65 (10.4%)	
** **	Lower 1/3	21 (70.0%)	464 (74.2%)	
cT	T1	15 (50.0%)	313 (50.1%)	0.511
** **	T2	5 (16.7%)	163 (26.1%)	
** **	T3	5 (16.7%)	85 (13.6%)	
** **	T4	5 (16.7%)	64 (10.2%)	
cN	N0	25 (83.3%)	514 (82.2%)	0.447
** **	N1	2 (6.7%)	78 (12.5%)	
** **	N2	2 (6.7%)	27 (4.3%)	
** **	N3	1 (3.3%)	6 (1.0%)	
cM	M0	29 (96.7%)	620 (99.1%)	0.155
** **	M1	1 (3.3%)	5 (0.8%)	

Data given as numbers (%) and means (±SD). Chi square test was used to evaluate between-group differences in categorical variables and a *p* value < 0.05 was deemed to indicate statistical significance.

TP, transplantation; BMI, Body mass index; PS, Performance status; ECOG, Eastern Cooperative Oncology Group; DM, diabetes mellitus.

**Table 2:** Postoperative outcomes of the patients who underwent gastric cancer surgery after transplantation and under immunosuppressant.

		TP group (N=30)	None TP group (N= 625)	P-value
Resection extent	Total gastrectomy	5 (16.7%)	123 (19.7%)	< 0.001
	Distal gastrectomy	21 (70.0%)	501 (80.2%)	
	Others	4 (13.3%)	1 (0.2%)	
LN dissection	D0/D1/D1+	14 (46.7%)	163 (26.1%)	0.013
	D2/D2+	16 (53.3%)	462 (73.9%)	
Operation time		174.86 (±68.5)	176.79 (±49.09)	0.842
EBL		94.00 (±87.2)	106.07 (±175.4)	0.732
Postoperative Hospital stays		11.48 (±6.3)	8.66 (±4.8)	0.003
Tumor size		3.35 (±3.3)	4.18 (±3.51)	0.141
Differentiation	Differentiated	20 (74.1%)	400 (64.2%)	0.294
	Undifferentiated	7 (25.9%)	223 (35.8%)	
Lymphatic invasion	No	13 (50.0%)	380 (60.8%)	0.270
	Present	13 (50.0%)	245 (39.2%)	
Vascular invasion	No	23 (88.5%)	530 (84.8%)	0.609
	Present	3 (11.5%)	95 (15.2%)	
Neural invasion	No	15 (60.0%)	494 (80.5%)	0.013
	Present	10 (40.0%)	120 (19.5%)	
Retrieved LNs		22.21 (±15.9)	43.98 (±17.55)	< 0.001
pT	T1	13 (44.8%)	408 (65.3%)	< 0.001
** **	T2	4 (13.8%)	58 (9.3%)	
** **	T3	2 (6.9%)	64 (10.2%)	
** **	T4	10 (33.4%)	95 (15.2%)	
pN	N0	13 (44.8%)	402 (64.8%)	< 0.001
** **	N1	3 (10.3%)	89 (14.2%)	
** **	N2	6 (20.7%)	59 (9.4%)	
** **	N3	6 (20.7%)	75 (12.0%)	
pM	M0	24 (80.0%)	606 (97.0%)	< 0.001
** **	M1	6 (20.0%)	19 (3.0%)	
p Stage	I	12 (40.0%)	408 (64.4%)	< 0.001
	II	4 (13.4%)	94 (15.0%)	
** **	III	8 (26.7%)	104 (16.6%)	
** **	IV	6 (20.0%)	19 (3.0%)	
Total Complication	No	21 (70.0%)	448 (71.7%)	0.954
	Present	9 (30.0%)	177 (28.4%)	

Data given as numbers (%) and means (±SD). Chi square test was used to evaluate between-group differences in categorical variables and a *p* value < 0.05 was deemed to indicate statistical significance.

TP, transplantation; EBL, estimated blood loss; LN, lymph node.

There were no differences in the preoperative clinical stages. However, the pathological stage was significantly advanced in the TP group; thus, we analyzed the stage up-migration rates and related factors. **Table 3** shows that the rates of up-migration from the clinical to the pathological stages (T, M, and final stages) were significantly higher in the TP group. Conversely, the down-migration rates of the T and final stages were significantly lower in the TP group (*p* = 0.022, *p* = 0.002, and *p* < 0.001, respectively). The up-migration rate for the N stage was also higher in the TP group, although this difference was not statistically significant (*p* = 0.286). Univariate and multivariate analyses revealed that tumor location and TP were significant risk factors for T stage up-migration (**Table 4**-**1**; hazard ratio [HR] = 0.484, *p* = 0.007 and HR = 2.870, *p* = 0.010, respectively). For N stage up-migration, only tumor differentiation was significant in the multivariate analysis (**Table 4**-**2**; HR = 0.661, *p* = 0.024). Age > 60 years and TP were significant factors for M stage up-migration (**Table 4**-**3**; HR = 3.792, *p* = 0.019 and HR = 8.253, *p* < 0.001, respectively). Final-stage up-migration risk factors were tumor location and TP in the univariate and multivariate analyses (**Table 4**-**4**; HR = 0.484, *p* = 0.007 and HR = 3.799, *p* < 0.001, respectively).

**Table 3:** Proportion of the stage migration from the Clinical stage to the Pathologic stage.

		TP group (N=30)	None TP group (N= 625)	P-value
**T stage**	Down (overestimation)	2 (6.7%)	138 (22.1%)	0.022
	Same	18 (60.0%)	394 (63.0%)	
	Up (underestimation)	10 (33.3%)	93 (14.9%)	
**N stage**	Down (overestimation)	1 (3.33%)	29 (4.6%)	0.286
	Same	16 (53.3%)	403 (64.5%)	
	Up (underestimation)	13 (43.3%)	193 (30.9%)	
**M stage**	Down (overestimation)	0	0	0.002
	Same	25 (83.3%)	607 (97.1%)	
	Up (underestimation)	5 (16.7%)	18 (2.9%)	
**Final stage**	Down (overestimation)	1 (3.33%)	65 (10.4%)	< 0.001
	Same	15 (50.0%)	443 (70.9%)	
	Up (underestimation)	14 (46.7%)	117 (18.7%)	

Data given as numbers (%). Chi square test was used to evaluate between-group differences in categorical variables and a *p* value < 0.05 was deemed to indicate statistical significance.

TP, transplantation.

**Table 4-1:** Univariate and Multivariate analysis for the up-stage migration associated factors; from the clinical T stage to the pathologic T stage.

		HR		95% CI			P-value	HR	95% CI				P-value
				lower	upper				lower			upper	
Age	< 60												
	≥ 60	1.239		0.804	1.909		0.332						
Sex	Male												
	Female	0.881		0.564	1.376		0.578						
Comorbidity	HTN	0.899		0.577	1.400		0.637						
	DM	1.513		0.895	2.559		0.122						
Smoking	Never												
	Quit	0.602		0.353	1.029		0.063						
	Current	0.934		0.571	1.528		0.786						
Alcohol	Never												
	Social	1.050		0.602	1.831		0.863						
	Heavy	0.840		0.470	1.500		0.556						
Tumor location	Upper 1/3												
	Middle 1/3	0.538		0.376	1.666		0.538						
	Lower 1/3	0.490		0.289	0.831		0.008	0.484	0.285			0.823	0.007
Differentiation	D												
	UD	0.898		0.574	1.404		0.637						
Immunosuppressant	No												
	Yes	2.860		1.298	6.305		0.009	2.870	1.291			6.379	0.010

HR, Hazard Ratio; CI, confidence interval; HTN, Hypertension; DM, diabetes mellitus; D, differentiated; UD, undifferentiated.

**Table 4-2:** Univariate and Multivariate analysis for the up-stage migration associated factors; from the clinical N stage to the pathologic N stage.

		HR	95% CI				P-value	HR	95% CI				P-value
			lower		upper				lower			upper	
Age	< 60												
	≥ 60	1.971	1.391		2.793		< 0.001						
Sex	Male												
	Female	0.912	0.645		1.290		0.602						
Comorbidity													
	HTN	1.386	0.987		1.947		0.059						
	DM	1.398	0.907		2.156		0.129						
Smoking	Never												
	Quit	0.723	0.483		1.083		0.115						
	Current	0.989	0.666		1.467		0.959						
Alcohol	Never												
	Social	0.781	0.539		1.131		0.191						
	Heavy	0.765	0.489		1.198		0.242						
Tumor location	Upper 1/3												
	Middle 1/3	0.974	0.505		1.878		0.937						
	Lower 1/3	0.970	0.611		1.539		0.897						
Differentiation	D												
	UD	0.666	0.465		0.953		0.026	0.661	0.462			0.946	0.024
Immunosuppressant	No												
	Yes	1.712	0.815		3.594		0.156						

HR, Hazard Ratio; CI, confidence interval; HTN, Hypertension; DM, diabetes mellitus; D, differentiated; UD, undifferentiated.

**Table 4-3:** Univariate and Multivariate analysis for the up-stage migration associated factors; from the clinical M stage to the pathologic M stage.

		HR		95% CI			P-value	HR			95% CI		P-value
				lower		upper					lower	upper	
Age	< 60												
	≥ 60	3.400		1.138		10.161	0.028	3.792			1.244	11.560	0.019
Sex	Male												
	Female	0.791		0.321		1.952	0.612						
Comorbidity													
	HTN	3.487		1.456		8.352	0.005						
	DM	1.460		0.530		4.023	0.464						
Smoking	Never												
	Quit	0.470		0.166		1.327	0.154						
	Current	0.392		0.127		1.211	0.104						
Alcohol	Never												
	Social	0.194		0.056		0.675	0.010						
	Heavy	0.487		0.159		1.485	0.206						
Tumor location	Upper 1/3												
	Middle 1/3	0.951		0.155		5.845	0.957						
	Lower 1/3	1.246		0.360		4.314	0.728						
Differentiation	D												
	UD	0.910		0.362		2.289	0.842						
Transplantation	No												
	Yes	6.744		2.317		19.633	0.000	8.253			2.730	24.949	< 0.001

HR: Hazard Ratio, CI: confidence interval, HTN; Hypertension, DM: diabetes mellitus, D; differentiated, UD; undifferentiated.

**Table 4-4:** Univariate and Multivariate analysis for the up-stage migration associated factors; from the clinical total stage to the pathologic total stage.

		HR		95% CI			P-value	HR		95% CI			P-value
				lower		upper				lower		upper	
Age	< 60												
	≥ 60	1.316		0.886		1.955	0.174						
Sex	Male												
	Female	0.983		0.659		1.468	0.935						
Comorbidity													
	HTN	1.427		0.966		2.108	0.074						
	DM	1.209		0.732		1.996	0.458						
Smoking	Never												
	Quit	0.600		0.377		0.955	0.031						
	Current	0.589		0.366		0.946	0.028						
Alcohol	Never												
	Social	0.723		0.474		1.102	0.131						
	Heavy	0.478		0.271		0.844	0.011						
Tumor location	Upper 1/3												
	Middle 1/3	0.946		0.486		2.165	0.946						
	Lower 1/3	0.916		0.539		1.558	0.747	0.484		0.285		0.823	0.007
Differentiation	D												
	UD	0.906		0.603		1.363	0.636						
Transplantation	No												
	Yes	2.870		1.291		6.379	0.010	3.799		1.804		8.002	< 0.001

HR, Hazard Ratio; CI, confidence interval; HTN, Hypertension; DM, diabetes mellitus; D, differentiated; UD, undifferentiated.

Propensity score matching was performed to compensate for the clinical differences in factors affecting postoperative outcomes and to compare surgical safety. Age, sex, comorbidities, tumor location, smoking/alcohol status, and clinical/pathological stages were balanced between the two groups after matching, whereas BMI and ECOG status remained different (**Table 5**, *p* = 0.002 and *p* = 0.037, respectively). No significant differences in postoperative outcomes were observed between the two groups, including operating time, excessive blood loss. In terms of postoperative recovery, the total incidence of complications (30.0 *vs* 40.0%, *P* = 0.417) and of severe Clavien–Dindo III-V complications (16.7 *vs* 13.4%, *P* = 0.417) did not differ significantly between the two groups (**Table 6**). There was no significant difference between the groups in rates of intra-abdominal infection, anastomosis leakage or wound infection, which could potentially be affected by immunosuppressants, (**Table 6**, 10 *vs* 13.3%), or in respiratory complications (**Table 6**, 3.3 *vs* 6.6%). We also analyzed whether there was a relationship between the type of immunosuppressant prescribed and the complication rate or type of complication, but no statistically significant results were found (data not shown).

**Table 5:** Preoperative clinical characteristics of the patients who underwent gastric cancer surgery after transplantation and under immunosuppressant (After 1:1 Propensity score matching).

		TP group (N=30)	None TP group (N= 30)	P-value
Age		59.23 (±12.62)	64.03 (±10.70)	0.118
Sex	M	20 (66.7%)	13 (43.3%)	0.069
	F	10 (33.3%)	17 (56.7%)	
BMI		20.87 (3.24)	23.62 (3.05)	0.002
PS (ECOG)	0	5 (16.7%)	14 (46.7%)	0.037
	1	22 (73.3%)	11 (36.7%)	
	2	2 (6.7%)	3 (110.0%)	
	3	2 (6.7%)	2 (6.7%)	
Type of TP	Kidney	19 (63.3%)		
	Liver	4 (13.3%)		
	Heart	1 (3.3%)		
	Bone marrow	6 (20.0%)		
Type of immunosuppressant	CNI	30 (100%)		
	m-TOR inhibitor	2 (6.7%)		
	Steroids	19 (63.3%)		
Comorbidity	none			
	Hypertension	18 (60.0%)	16 (53.3%)	0.602
	DM	16 (53.3%)	12 (40.0%)	0.301
	Pulmonary	2 (6.7%)	1 (3.3%)	0.554
	others	29 (96.7%)	27 (90.0%)	0.301
Smoking	None	27 (90.0%)	28 (93.3%)	0.839
	Ex-smoker	2 (6.7%)	1 (3.3%)	
	Current smoker	1 (3.3%)	1 (3.3%)	
Alcohol	None	29 (96.7%)	28 (93.3%)	0.554
	Social drinking	1 (3.3%)	2 (6.7%)	
	Heavy drinking	1 (0%)	1 (0%)	
Cancer location	Upper 1/3	4 (13.3%)	3 (10.0%)	0.656
	Middle 1/3	5 (16.7%)	3 (10.0%)	
	Lower 1/3	21 (70.0%)	24 (80.0%)	
cT	T1	15 (50.0%)	12 (40.0%)	0.860
	T2	5 (16.7%)	7 (23.3%)	
	T3	5 (16.7%)	5 (16.7%)	
	T4	5 (16.7%)	6 (20.0%)	
cN	N0	25 (83.3%)	25 (83.3%)	0.753
	N1	2 (6.7%)	2 (6.7%)	
	N2	2 (6.7%)	3 (10.0%)	
	N3	1 (3.3%)	0 (0%)	
cM	M0	29 (96.7%)	30 (100%)	0.313
	M1	1 (3.3%)	0 (0%)	

Data given as numbers (%). Chi square test was used to evaluate between-group differences in categorical variables and a *p* value < 0.05 was deemed to indicate statistical significance.

TP, transplantation; BMI, Body mass index; PS, Performance status; ECOG, Eastern Cooperative Oncology Group; DM, diabetes mellitus .

**Table 6:** Postoperative outcomes of the patients who underwent gastric cancer surgery after transplantation and under immunosuppressants (After 1:1 Propensity matching).

		TP group (N=30)	None TP group (N= 30)	P-value
Resection extent	Total gastrectomy	5 (16.7%)	3 (10.0%)	0.258
	Distal gastrectomy	21 (70.0%)	26 (86.7%)	
	Others	4 (13.3%)	1 (3.3%)	
LN dissection	D0/D1/D1+	14 (46.7%)	9 (30.0%)	0.184
	D2/D2+	16 (53.3%)	21 (70.0%)	
Operation time		174.86 (±68.57)	163.93 (±43.97)	0.470
EBL		94.00 (±87.23)	141.83 (±213.44)	0.299
Postoperative Hospital stays		11.48 (±6.35)	8.90 (±5.07)	0.089
Total Complication	No	21 (70.0%)	18 (60.0%)	0.417
	Present	9 (30.0%)	12 (40.0%)	
	pulmonary	2 (22.2%)	4 (33.3%)	
	Anastomosis leakage	0	0	
	Duodenal stump leakage	2 (22.2%)	1 (8.4%)	
	Postop bleeding	3 (33.3%)	0	
	Wound infection	1 (11.1%)	3 (25.0%)	
	other	4 (44.4%)	4 (33.3%)	
C-D III-V	No	25 (83.3%)	26 (86.6%)	0.676
	Present	5 (16.7%)	4 (13.4%)	
pStage	I	12 (40.0%)	17 (56.7%)	0.662
	II	4 (13.4%)	4 (13.3%)	
** **	III	8 (26.7%)	7 (23.4%)	
** **	IV	6 (20.0%)	2 (6.7%)	
Tumor size		3.35 (±3.37)	4.88 (±4.37)	0.144
Differentiation	Differentiated			
** **	Undifferentiated			
Lymphatic invasion	No	13 (50.0%)	15 (50.0%)	0.999
** **	Present	13 (50.0%)	15 (50.0%)	
Vascular invasion	No	23 (88.5%)	24 (80.0%)	0.390
** **	Present	3 (11.5%)	6 (20.0%)	
Neural invasion	No	15 (60.0%)	19 (67.9%)	0.552
** **	Present	10 (40.0%)	9 (32.1%)	
Retrieved LNs		22.21 (±15.99)	39.53 (±16.25)	< 0.001

Data given as numbers (%). Chi square test was used to evaluate between-group differences in categorical variables and a *p* value < 0.05 was deemed to indicate statistical significance.

TP, transplantation; LNs, lymph nodes; EBL, estimated blood loss; C-D, Clavian-Dindo.

Among the total 655 patients, Stage 2 and 3 patients were 198 and 12 in the non-TP and TP groups, respectively. There was no significant difference in the proportion of adjuvant chemotherapy between the two groups (48.48% *vs* 58.33%, respectively. P =0.352). In terms of overall survival, the TP group showed significantly worse prognosis in stages I, II, and IV (*P* < 0.001*, P* = 0.039 and 0.007, respectively, **Supplementary Figure 2**). In disease free survival, the TP group showed worse prognosis in stage I, and there was no significant difference in stage II and III (*P* < 0.001*, P* = 0.562 and 0.083, respectively, **Supplementary Figure 3**). In a review of patients who had recurrence in Stage I, there were three patients in the TP group. Despite securing safe pathologic margins of more than 2 cm, recurrence occurred in all patients at the remnant stomach. And recurrence occurred within 1 year in two patients, raising the possibility of synchronous cancer.

## Discussion

Many studies have shown that the use of immunosuppressive drugs decreases the immune surveillance that suppresses host cancer and cancer-related viral proliferation (12), thereby increasing cancer incidence and decreasing natural killer cell activity (13), which is closely related to cancer occurrence. Regardless of the immunosuppressive effect, immunosuppressants induce cancer by producing cytokines (14), such as transforming growth factor-β and vascular endothelial growth factor (VEGF); in contrast, drugs such as azathioprine induce cancer by interfering with DNA damage repair (15). Although there is minimal evidence that any specific drug causes a particular cancer, there are reports regarding the associations of various immunosuppressants with cancer.

Calcineurin inhibitors, which are mainly used after liver and kidney transplantation, contribute to cancer development by increasing cytokines such as transforming growth factor-β, VEGF, and interleukin-6 (14). Conversely, mammalian target of rapamycin (mTOR) inhibitors, such as sirolimus, reduce the incidence of cancer by suppressing p70 S6K, interleukin-10, and cyclin; downregulating VEGF; and inducing apoptosis (4). Mycophenolate mofetil (MMF) inhibits inosine monophosphate dehydrogenase and blocks purine biosynthesis to produce an immunosuppressive effect. MMF can reduce the risks of some cancers that contain large amounts of inosine monophosphate dehydrogenase (16). However, the incidence of cancer was reportedly high when immunosuppressants were combined with MMF (16), so additional basic research is needed concerning specific immunosuppressants and the mechanism of cancer development.

Gastric cancer is associated with *Helicobacter pylori* and Epstein–Barr virus infection (17–19). The rate of *de novo* gastric cancer after transplantation is much higher in Korea than in Western countries (7, 20). Furthermore, the *H*. *pylori* infection rate is nearly twice as high in kidney transplant patients, which may partially explain the high incidence of gastric cancer among transplant patients in Korea (21). This pattern also appears in human papillomavirus-related cervical cancer (22). The human papillomavirus infection rate is high in Korea; the incidence of cervical cancer in transplant patients is also high (23).

Few studies have investigated the short- or long-term outcomes of gastric cancer surgery after transplantation. One study revealed similar perioperative and short-term outcomes of gastric cancer surgery between patients with and without a history of organ transplantation, including kidney, liver, and heart transplantation (24). However, the transplant patient group had worse long-term survival outcomes, compared with the non-transplant group. This finding is consistent with our results; patients taking immunosuppressive drugs safely underwent radical gastrectomy without any short-term postoperative differences.

Notably, many patients had a more advanced form of disease than had been determined preoperatively, and the use of immunosuppressive drugs was a significant factor in up-migration. This novel finding will be very important in determining the treatment plan when cancer occurs in transplant patients. A possible explanation for this finding is that the immunosuppressive drug itself interfered with the cancer surveillance system. As a lymph node enlarges, inflammation develops in the peri-tumor environment, ulcers occur, and the gastric wall thickens, enabling estimation of the clinical stage. The stage may have been underestimated because a sufficient anti-cancer immune reaction did not occur. Accordingly, patients taking immunosuppressants require regular follow-up and cancer screening. One study showed a survival benefit of regular follow-up after transplantation. For gastric cancer patients, the regular follow-up group had better 2-year survival with a higher rate of early gastric cancer detection (survival 93.1% *vs*. 33.3%, *p* = 0.006) (25). Therefore, active *H*. *pylori* eradication treatment would be helpful, based on regular *H*. *pylori* testing and endoscopy screening in transplant patients.

Some transplant patients at advanced cancer stages may not receive adjuvant chemotherapy because of concerns about graft failure during the use of chemotherapy drugs that could adversely affect the function of the transplanted graft. Even after the start of chemotherapy, the compliance and completion rates may be lowered depending on graft function, so complete surgical treatment should be considered more important than in the general patient population. When early gastric cancer is clinically diagnosed, an appropriate margin should be secured and sufficient lymph node dissection (such as D2 dissection) should be performed, considering the possibility of an advanced tumor and lymph node metastasis. Our study results showed that there was no difference in the proportion of adjuvant chemotherapy between the groups. Although there was no statistical difference in the recurrence rate between the two groups, the TP group showed a worse prognosis in terms of overall survivals, it may have been due to causes other than cancer recurrence. And this suggests that aggressive treatment with curative intent for gastric cancer may be effective in systemic control of gastric cancer. However, the high rate of recurrence in the remnant stomach among stage I patients in the TP group shows how important it is to decide on curative surgery in patients preoperatively diagnosed with early gastric cancer.

This study had several limitations. First, it used retrospective data for a small number of patients with histories of organ transplantation. Second, there was no information concerning *H*. *pylori* or Epstein–Barr virus infection status which could have been influenced to gastric cancer development. Third, the cohort of patients with prior history of transplantation was obtained from 1990-2021 spanning over 31 year when the method and quality of clinical staging has likely improved over the period of time. Therefore, this includes the possibility that it may have contributed to migration in the perioperative stage. Fourth, a history of organ transplantation might bias analysis of a patient’s postoperative safety. However, the important point in this analysis is that the effect of the immunosuppressants themselves was considered the main factor, rather than the type of transplant. Kidney transplantation involves transplanting a kidney into the retroperitoneum of the right lower quadrant of the abdomen; the surgical field is different from that of stomach cancer surgery and has little effect in most cases. When performing stomach cancer surgery, a history of liver transplantation is probably the biggest obstacle. This analysis included four liver transplant patients, and among them only two cases experienced minor complications—a minor wound infection and gastric stasis treated with conservative care—and there were no severe complications. If more patients can be registered in the future, subgroup analysis for each transplant type will be necessary.

Many studies have shown that the systemic immune capacity determines the prognosis of cancer treatment, although no prior study has examined patients taking immunosuppressive drugs; research is needed concerning ways to increase the effect of systemic therapy including for adjuvant or palliative purpose. However, a strength of our study is that it is the first to reveal the possibility of the stage being underestimated preoperatively, based on the hypothesis that taking immunosuppressants does not simply increase the incidence of *de novo* cancer, but also affects the treatment plan and perioperative procedures. This will serve as a cornerstone for examining the long-term impact of active treatment on the oncologic outcomes of transplant patients, confirming postoperative safety.

In conclusion, radical gastrectomy can be a safe oncological procedure for gastric cancer patients who are taking immunosuppressant agents after transplantation. Considering the immunosuppressed condition of those patients and the possibility of under-diagnosing gastric cancer, early detection with endoscopic screening and treatment with curative intent are needed.

## Data availability statement

All data and materials are available upon request. Anyone who wants the data should contact YJ.

## Ethics statement

The studies involving humans were approved by Institutional Review Board of the College of Medicine, Catholic University of Korea (KC22RISI0943). The studies were conducted in accordance with the local legislation and institutional requirements. Written informed consent for participation was not required from the participants or the participants’ legal guardians/next of kin in accordance with the national legislation and institutional requirements.

## Author contributions

JL: Investigation, Writing – original draft. SJK: Data curation, Formal Analysis, Investigation, Writing – review & editing. HS: Conceptualization, Investigation, Methodology, Writing – review & editing. HL: Conceptualization, Methodology, Supervision, Writing – review & editing. SGK: Conceptualization, Methodology, Supervision, Writing – review & editing. KJ: Data curation, Investigation, Methodology, Supervision, Writing – review & editing. KS: Conceptualization, Investigation, Methodology, Supervision, Writing – review & editing. YJ: Conceptualization, Supervision, Writing – review & editing, Writing – original draft.
