# Supplementary material for: Radical gastrectomy is safe for treatment of gastric cancer patients on immunosuppressive drugs after organ transplantation
Source: Front Oncol. 2024 Jan 10;13:1264628. doi: 10.3389/fonc.2023.1264628 (PMC10807791; doi:10.3389/fonc.2023.1264628)

**Supplementary Table 1. Preoperative clinical characteristics of the patients who underwent transplantation**

|  |  |  | N= 54 |
| --- | --- | --- | --- |
| Age |  |  | 58.25 (±1.47) |
| Sex | Male |  | 39 (72.2%) |
|  | Female |  | 15 (27.7%) |
| BMI |  |  | 21.22 (±0.46) |
| PS (ECOG) | 0 |  | 8 (14.8%) |
|  | 1 |  | 35 (64.8%) |
|  | 2 |  | 8 (14.8%) |
|  | 3  4 |  | 2 (3.7%)  1 (1.8%) |
| Comorbidity | none |  | 11 (20.3%) |
|  | Hypertension |  | 30 (55.5%) |
|  | DM |  | 16 (29.6%) |
|  | Pulmonary |  | 6 (11.1%) |
| Smoking | None |  | 41 (75.9%) |
|  | Ex-smoker |  | 4 (7.4%) |
|  | Current smoker |  | 3 (5.5%) |
| Alcohol | None |  | 29 (53.7%) |
|  | Social drinking |  | 16 (29.6%) |
|  | Heavy drinking |  | 3 (5.5%) |
| Transplantation | Kidney |  | 39 (72.2%) |
|  | Liver |  | 4 (7.4%) |
|  | Bone marrow |  | 10 (18.5%) |
|  | Heart |  | 1 (1.8%) |
| Immunosuppressant | No |  | 9 (16.6%) |
|  | Yes | 1 | 26 (48.1%) |
|  |  | ≥2 | 19 (35.1%) |
| Tumor location | Lower |  | 35 (64.8%) |
|  | Middle |  | 8 (14.8%) |
|  | Upper |  | 7 (12.8%) |
| Clinical stage | cT1 |  | 24 (44.4%) |
|  | cT2-4 |  | 23 (42.5%) |
|  | Unknown |  | 7 (12.9%) |

Data given as numbers (%) and means (±SD). BMI: Body mass index, PS: Performance status, ECOG: Eastern Cooperative Oncology Group, DM: diabetes mellitus

**Supplementary Figure 1. Algorithm for patient inclusion and exclusion**


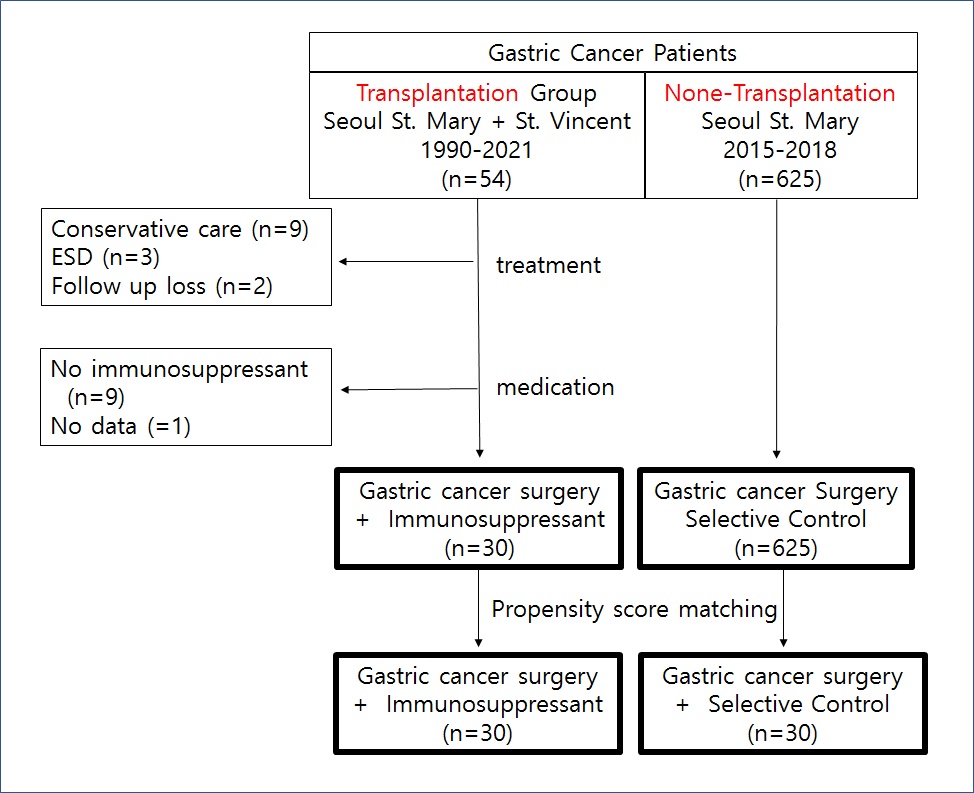


**Supplementary Figure 2. Overall survival by TP status. A) pathologic stage I (P < 0.001), B) stage II (P = 0.039), C) stage III (P = 0.660), D) stage IV (P = 0.007).**


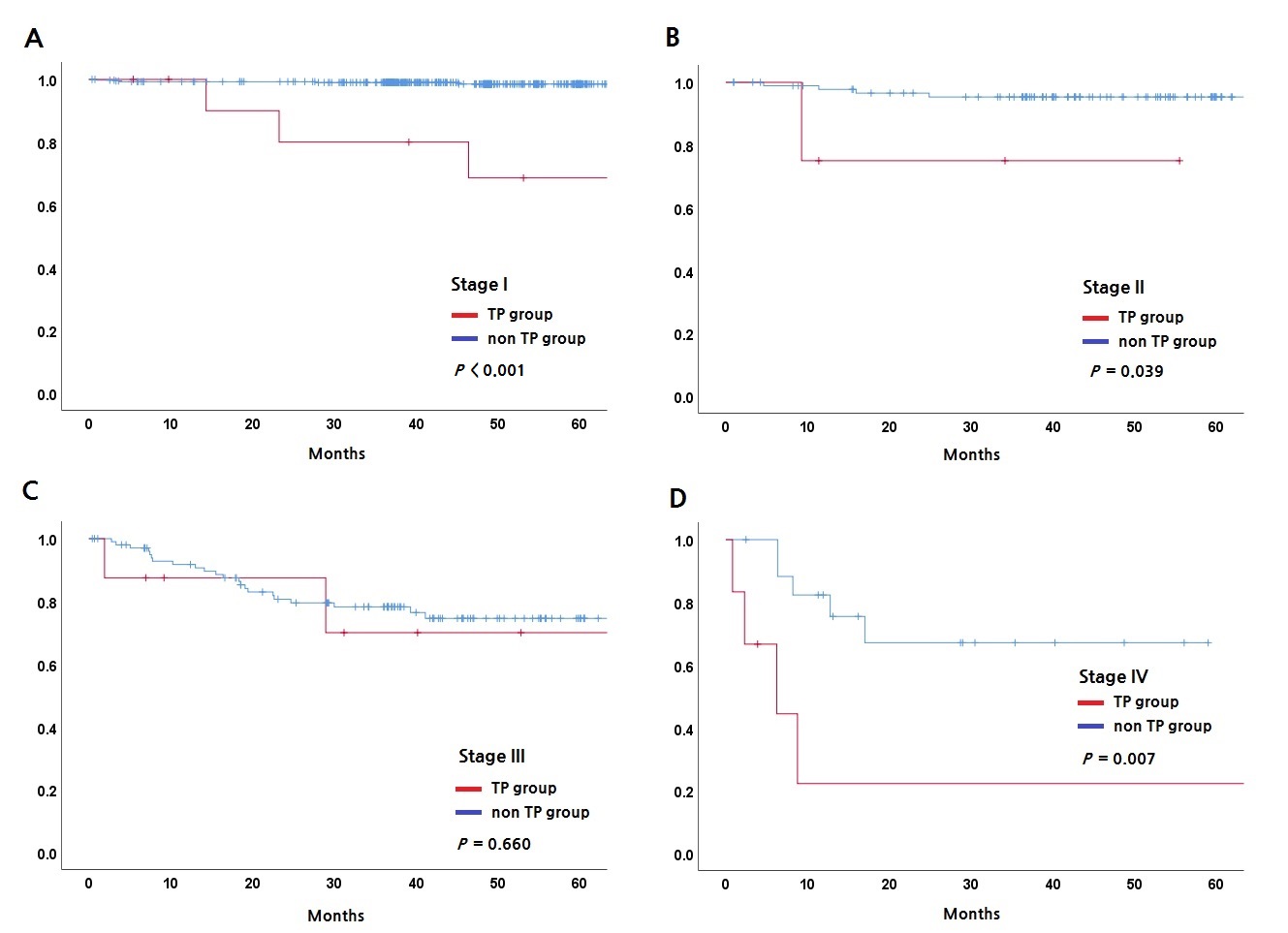


**Supplementary Figure 3. Disease free survival by TP status. A) pathologic stage I (P < 0.001), B) stage II (P = 0.562), C) stage III (P = 0.083)**


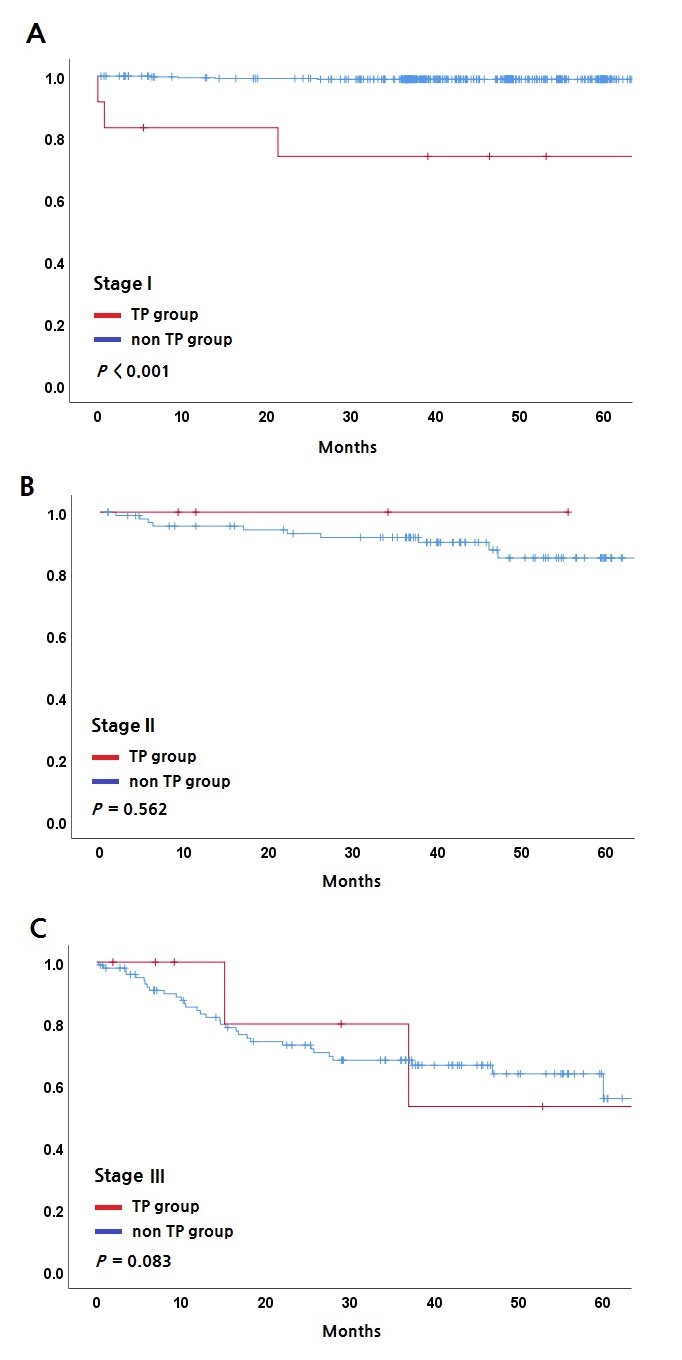

Supplement: Supplementary file 1 [file DataSheet_1.docx]
